# Variation in Soil Bacterial and Fungal Diversity, Community Composition, and Co-Occurrence Patterns Across Nearshore Islands in the Northern South China Sea

**DOI:** 10.3390/microorganisms14081838

**Published:** 2026-08-19

**Authors:** Jian Gong, Yechen Qiao, Rui Pan, Hu Du, Xionghui Liao, Wanxia Peng

**Affiliations:** 1 Guangxi Key Laboratory of Environmental Processes and Remediation in Ecologically Fragile Regions, Guangxi Normal University, Guilin 541006, China; 15731930730@163.com (J.G.); 18014657118@163.com (Y.Q.); 2 Institute of Subtropical Agriculture, Chinese Academy of Sciences, Changsha 410125, China; pan__r@163.com (R.P.); hudu@isa.ac.cn (H.D.); xhliao@isa.ac.cn (X.L.); 3 Huanjiang Observation and Research Station for Karst Ecosystem, Guangxi Key Laboratory of Karst Ecological Processes and Services, Institute of Subtropical Agriculture, Chinese Academy of Sciences, Huanjiang 547100, China; 4 Guangxi Key Laboratory of Forest Ecology and Conservation, State Key Laboratory for Conservation and Utilization of Subtropical Agro-Bioresources, School of Forestry, Guangxi University, Nanning 530004, China

**Keywords:** microbial island biogeography, environmental heterogeneity, soil salinity, Bray–Curtis dissimilarity, co-occurrence associations, nearshore islands

## Abstract

Soil microbial biogeography on nearshore islands remains less well understood than that on remote archipelagos, particularly when bacterial and fungal diversity, community composition, and co-occurrence associations are evaluated together. We characterized bacterial 16S rRNA gene and fungal ITS communities in 36 topsoil samples collected from five nearshore islands in the northern South China Sea and examined their associations with plant diversity, soil physicochemical properties, and climatic and geographic conditions. Bacterial Shannon diversity, ACE richness, and Simpson diversity differed significantly among islands, as did fungal ACE richness, whereas fungal Shannon and Simpson diversity showed no significant differences. Bray–Curtis-based analyses revealed island-associated differentiation in bacterial community composition and fungal community composition, although multivariate dispersion also differed among islands. Sample-level subnetworks derived from an integrated co-occurrence network showed that the number of bacteria–bacteria edges differed significantly among islands, whereas the numbers of bacteria–fungi and fungi–fungi edges did not. Exploratory Spearman correlation analyses and variation partitioning indicated that microbial properties were more broadly associated with soil physicochemical properties and climatic–geographic conditions than with plant diversity. By evaluating both microbial kingdoms within the same field campaign, this study identifies partly distinct bacterial and fungal biogeographic and co-occurrence patterns and provides a baseline for future microbial biodiversity monitoring on nearshore islands.

## 1. Introduction

Oceanic islands play an important role in maintaining biodiversity and regulating ecosystem functions due to their unique geographic isolation and environmental heterogeneity [1,2,3,4,5]. They are widely regarded as natural systems for studying the assembly and functioning of soil microbial communities, owing to their relatively simple biotic composition and diverse environmental gradients [6,7,8,9]. Soil microorganisms, such as bacteria and fungi, are key drivers of nutrient cycling and vegetation stability on oceanic islands [10,11,12,13]. They mediate organic matter decomposition and nutrient transformation and form close symbiotic associations with plants [14,15]. However, the diversity and community composition of soil bacteria and fungi, as well as their co-occurrence patterns, vary across oceanic islands [6,7,12]. These patterns may be associated with multiple environmental factors. Therefore, clarifying the environmental conditions associated with these patterns is important for understanding the maintenance and development of oceanic island ecosystems.

Bacterial and fungal diversity, community composition, and co-occurrence networks are associated with plant diversity, geographic location, and climatic factors [16,17]. Generally, plant communities regulate microbial substrate availability and niche differentiation through litter inputs, root exudates, and resource heterogeneity [8,18,19,20,21]. Recent studies have shown that increased plant diversity can enhance positive associations within rhizosphere microbial networks and improve microbial carbon-use efficiency [22,23,24]. Climatic factors, such as temperature, precipitation, and soil moisture, directly influence microbial metabolism and dispersal [16,25,26,27]. Previous studies have found that extreme climatic events, including drought, flooding, freezing injury, and heat stress, generally induce substantial shifts in soil microbial communities, although the magnitude of these responses is jointly regulated by soil physicochemical properties and local climate [28,29,30]. Under drought conditions, fungi often maintain higher persistence and activity than bacteria [31,32,33], indicating differential responses of bacterial and fungal communities to water stress. Overall, these findings suggest that plant, soil, and climatic conditions may be jointly associated with bacterial and fungal communities.

Oceanic islands differ from mainland ecosystems in plant diversity, soil physicochemical properties, and climatic–geographic conditions [1,3]. Larger oceanic islands with more complex vegetation generally provide a greater diversity of ecological niches and higher carbon inputs, which can enhance bacterial diversity and strengthen microbial associations [6,14,34,35]. In contrast, oceanic islands with simpler vegetation may reduce substrate heterogeneity, thereby limiting microbial richness [36]. Previous studies on tropical oceanic islands in the South China Sea have shown that bacterial diversity is closely linked to plant species richness, whereas fungal richness is more strongly regulated by climate, oceanic islands area, soil pH, and plant traits [9]. Soil physicochemical properties also play key roles in shaping microbial communities on oceanic islands [37]. Higher nitrogen and potassium availability can increase bacterial richness and promote positive microbial associations, whereas elevated salinity suppresses fungal richness and reduces the abundance of salt-tolerant taxa [38,39,40]. Climatic stress further influences microbial community assembly across oceanic islands [41,42,43]. Strong winds, high evaporation, and prolonged sunshine duration reduce soil moisture, while temperature differences among oceanic islands reinforce these effects [44]. Collectively, these climatic constraints may be associated with lower bacterial and fungal richness. Consequently, it remains unclear whether bacterial and fungal communities exhibit comparable response patterns along environmental gradients on nearshore islands.

To address this gap, we selected five representative nearshore islands in the northern South China Sea and compared the diversity, community composition, and co-occurrence network characteristics of soil bacterial and fungal communities. We further evaluated the fractions of microbial variation associated with plant diversity, soil physicochemical properties, and climatic–geographic factors. We hypothesized that (1) bacterial and fungal diversity, community composition, and co-occurrence network characteristics would differ among islands and (2) bacterial and fungal communities would show partly distinct associations with plant diversity, soil salinity and nutrient availability, and climatic–geographic conditions, including temperature, evaporation, sunshine duration, island area, and isolation.

## 2. Materials and Methods

### 2.1. Study Area

**Figure 1:**
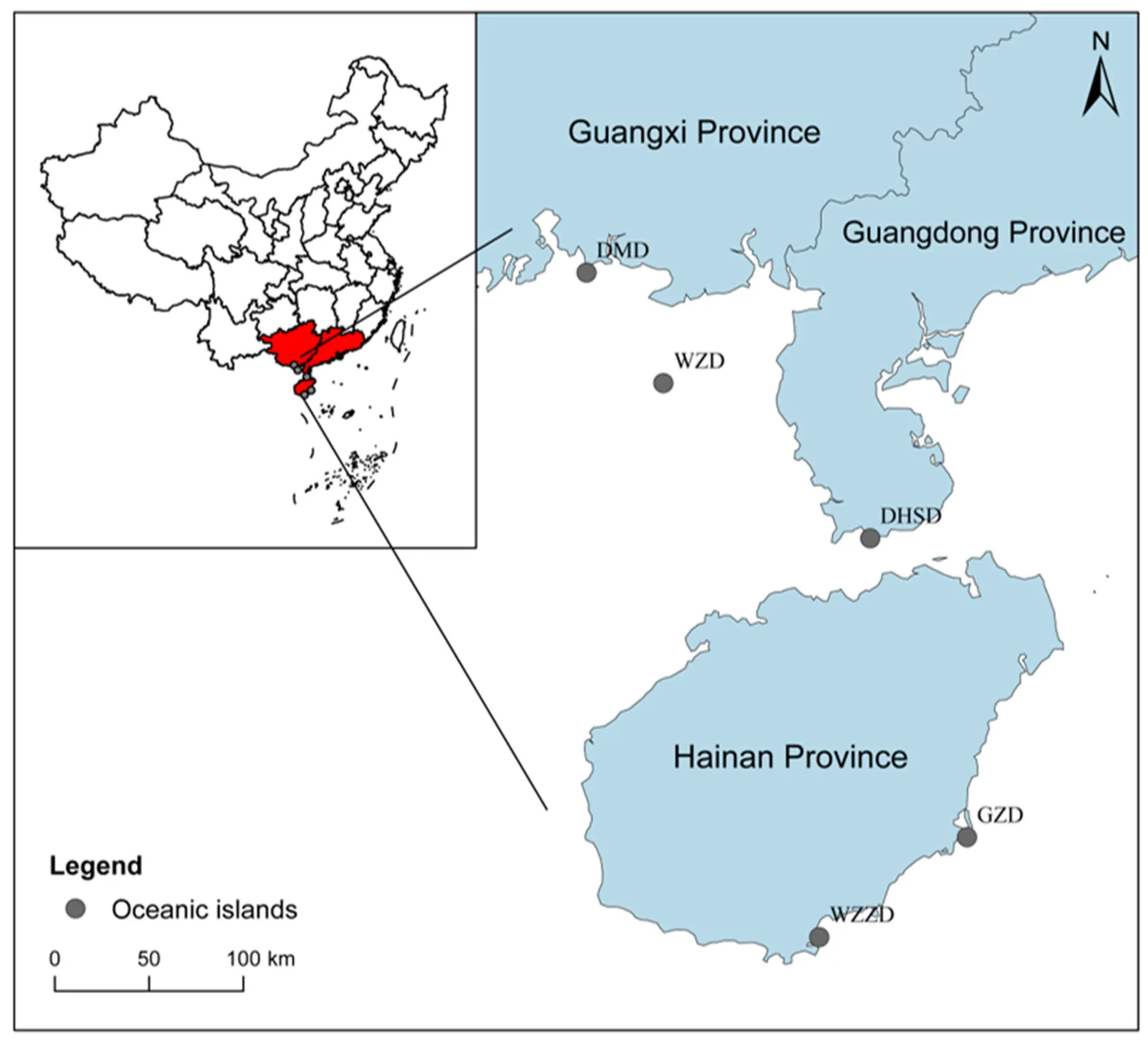
Geographic locations of the five oceanic islands in the northern South China Sea. DHSD, Da-Han-San-Dun Island; DMD, Da-Miao-Dun Island; GZD, Gan-Zhe Island; WZD, Wei-Zhou Island; WZZD, Wu-Zhi-Zhou Island.

The study area comprised five nearshore islands in the northern South China Sea, China (18°18′ N–21°35′ N, 108°44′ E–109°45′ E) (Figure 1). Among them, Da-Miao-Dun Island, Gan-Zhe Island, and Wu-Zhi-Zhou Island are bedrock islands, Da-Han-San-Dun Island is an accumulation island, and Wei-Zhou Island is a volcanic island. Oceanic island area ranged from 0.62 to 24.74 km^2^, and the distance from the mainland ranged from 0.84 to 37.16 km. The study region has a subtropical monsoon climate, with a rainy season from June to September and a dry season from October to the following May. Mean annual temperature and precipitation ranged from 22 to 25.7 °C and from 1347.5 to 2154 mm, respectively. More details on the climatic and geographic characteristics of the five oceanic islands are shown in Table S1. Da-Miao-Dun Island, Da-Han-San-Dun Island, and Gan-Zhe Island are uninhabited and subject to relatively low human disturbance. *Casuarina equisetifolia* is the dominant species on Da-Miao-Dun Island and Da-Han-San-Dun Island, whereas Gan-Zhe Island is mainly covered by shrub communities dominated by *Scaevola taccada* and *Pandanus tectorius*. In contrast, Wei-Zhou Island and Wu-Zhi-Zhou Island have small resident populations and experience relatively greater human disturbance, with more complex vegetation structures, including tree-layer species such as *Casuarina equisetifolia*, *Leucaena leucocephala*, *Acacia confusa*, and *Hydnocarpus hainanensis*; the shrub-layer species *Pandanus tectorius*; and herb-layer species such as *Imperata cylindrica* and *Ipomoea pes-caprae*.

### 2.2. Soil Sampling and Plant Community Surveys

Plant surveys and soil sampling were conducted in November 2018. Thirty-six plots were allocated among the five islands according to island area and the distribution of representative vegetation types. The numbers of plots on Da-Miao-Dun, Wei-Zhou, Da-Han-San-Dun, Gan-Zhe, and Wu-Zhi-Zhou Islands were 3, 12, 3, 6, and 12, respectively. Plot size was determined by the dominant vegetation type: 20 m × 20 m for tree-dominated communities, 10 m × 10 m for shrub-dominated communities, and 1 m × 1 m for herb-dominated communities. In tree-dominated plots, all trees with a diameter at breast height ≥ 1 cm were recorded. In shrub-dominated plots, four 2 m × 2 m subquadrats were surveyed for species identity, clump number, height, and basal diameter. In herb-dominated plots, three 1 m × 1 m subquadrats were surveyed for species identity, clump number, coverage, and mean height.

Eight soil cores were randomly collected from the 0–10 cm layer of each plot after removing surface litter and were combined into one composite sample. Each composite sample was divided into separate portions for physicochemical and molecular analyses. The physicochemical portion was air-dried, ground, and sieved. The molecular subsample was stored at −60 °C until DNA extraction from fresh soil.

Plant diversity was characterized using the Shannon–Wiener diversity index, species richness, the Simpson index, and Pielou evenness. Species richness was defined as the number of plant species recorded in each plot. The indices were calculated at the plot level using abundance or cover records corresponding to the dominant vegetation type represented by each plot.

### 2.3. Analysis of Soil Physicochemical Properties

Soil pH was measured using a pH meter after shaking a soil–water suspension at a ratio of 1:2.5 (*w*/*v*) for 30 min. Soil bulk density was determined using the cutting-ring method. Soil organic carbon was measured by the dichromate oxidation method. Soil total nitrogen and available nitrogen were determined by ultraviolet spectrophotometry following Kjeldahl digestion and extraction with NaOH (1.0 mol L^−1^), respectively. Soil total potassium and available potassium were measured using atomic absorption spectrophotometry after digestion with NaOH and extraction with NH_4_OAc (1.0 mol L^−1^), respectively. Soil total phosphorus and available phosphorus were determined by the molybdenum-antimony colorimetric method after digestion with HClO_4_-H_2_SO_4_ (1:10, *v*/*v*) and extraction with NaHCO_3_ (0.5 mol L^−1^), respectively. Soil salt content was measured using a conductivity meter.

### 2.4. Soil Microbial Analysis

Total soil DNA was extracted from 0.5 g of fresh soil using the FastDNA SPIN Kit for Soil (MP Biomedicals, Irvine, CA, USA) according to the manufacturer’s instructions. DNA quality was assessed by 1% agarose gel electrophoresis. For bacterial community analysis, the V3–V4 region of the 16S rRNA gene was amplified using the primer pair 338F (5′-ACTCCTACGGGAGGCAGCAG-3′) and 806R (5′-GGACTACHVGGGTWTCTAAT-3′) [45,46]. For fungal community analysis, the ITS1 region was amplified using the primer pair ITS1F (5′-CTTGGTCATTTAGAGGAAGTAA-3′) and ITS2R (5′-GCTGCGTTCTTCATCGATGC-3′) [47].

PCR was performed in a 20 μL reaction mixture containing 4 μL of 5× FastPfu buffer, 2 μL of 2.5 mM dNTPs, 0.8 μL each of the forward and reverse primers (5 μM), 0.4 μL of TransStart FastPfu DNA Polymerase (AP221-02; TransGen Biotech Co., Ltd., Beijing, China), 0.2 μL of bovine serum albumin, approximately 10 ng of template DNA, and sterile water to the final volume. Amplification was conducted using a GeneAmp 9700 thermal cycler (Applied Biosystems, Foster City, CA, USA). The amplification program consisted of an initial denaturation at 95 °C for 3 min, followed by 27 cycles for bacteria or 35 cycles for fungi at 95 °C for 30 s, 55 °C for 30 s, and 72 °C for 45 s, with a final extension at 72 °C for 10 min. Each sample was amplified in triplicate, and the three PCR products from the same sample were pooled. Pooled PCR products were checked by 2% agarose gel electrophoresis, purified using the AxyPrep DNA Gel Extraction Kit (Axygen Biosciences, Union City, CA, USA), eluted with Tris–HCl buffer, and quantified using the QuantiFluor-ST fluorescence system (Promega Corporation, Madison, WI, USA). Purified products were pooled at equimolar concentrations, and sequencing libraries were prepared using the TruSeq DNA Sample Preparation Kit (Illumina, Inc., San Diego, CA, USA). Sequencing was performed by Shanghai Majorbio Bio-pharm Technology Co., Ltd. (Shanghai, China) on an Illumina HiSeq platform (Illumina, Inc., San Diego, CA, USA) using a paired-end 250-bp strategy.Following quality filtering and read assembly, high-quality bacterial 16S rRNA and fungal ITS sequences were dereplicated, and singleton sequences were removed. Sequences were clustered into operational taxonomic units (OTUs) at 97% sequence similarity using UPARSE (version 7.0.1090; http://drive5.com/uparse/), with chimeric sequences removed during clustering. All high-quality sequences were subsequently mapped to the representative OTU sequences at a similarity threshold of 97% to generate the OTU tables. Representative bacterial and fungal OTU sequences were taxonomically classified using the RDP Classifier (version 2.11; http://sourceforge.net/projects/rdp-classifier/) with a confidence threshold of 0.7 against the SILVA database (Release 138; http://www.arb-silva.de/) and the UNITE database (Release 8.0; http://unite.ut.ee/), respectively. Prokaryotic phylum names reported in the text and figures were updated according to Oren and Garrity [48]. Alpha-diversity indices were calculated from the resulting OTU tables.

### 2.5. Statistical Analysis

All statistical analyses were conducted in R (version 4.5.0; R Foundation for Statistical Computing, Vienna, Austria). Before one-way analysis of variance (ANOVA), residual normality and homogeneity of variance were assessed using Shapiro–Wilk and median-centered Levene tests, respectively. Differences in plant diversity, soil physicochemical properties, microbial alpha diversity, and sample-level network node and edge variables among islands were evaluated using one-way ANOVA, followed by Duncan’s multiple-range test when *p* < 0.05; omega-squared (ω^2^) was calculated for microbial alpha-diversity comparisons. Non-metric multidimensional scaling (NMDS) was performed from Bray–Curtis dissimilarities using metaMDS in the vegan package, and differences in bacterial and fungal community composition were tested using adonis2 with 9999 permutations. Multivariate dispersion was assessed using betadisper and a 9999-permutation permutest. Associations between environmental predictors and microbial variables were evaluated using two-sided Spearman rank correlations (psych::corr.test, method = “spearman”, adjust = “none”). No multiple-testing correction was applied to these 494 exploratory correlations. One integrated bacterial–fungal co-occurrence network was inferred from all 36 samples using WGCNA. OTUs with a summed relative abundance greater than 0.02 were retained, and network edges were defined by Spearman correlations with |ρ| > 0.6 and Benjamini–Hochberg-adjusted *p* < 0.01. Sample-level subnetworks containing more than five nodes were extracted from the integrated network for calculation of node and edge metrics, and the network was visualized in Gephi (version 0.9.2). Variation partitioning analysis was used to quantify the independent and shared fractions of bacterial and fungal variation associated with plant diversity, soil physicochemical properties, and climatic–geographic conditions, and multicollinearity among soil and plant-diversity variables was evaluated separately using variance inflation factors. Climatic–geographic variables were treated as an island-level variable group because they were represented by only five island-level combinations.

## 3. Results

### 3.1. Differences in Soil Physicochemical Properties, Plant Diversity, and Climatic–Geographic Factors

**Figure 2:**
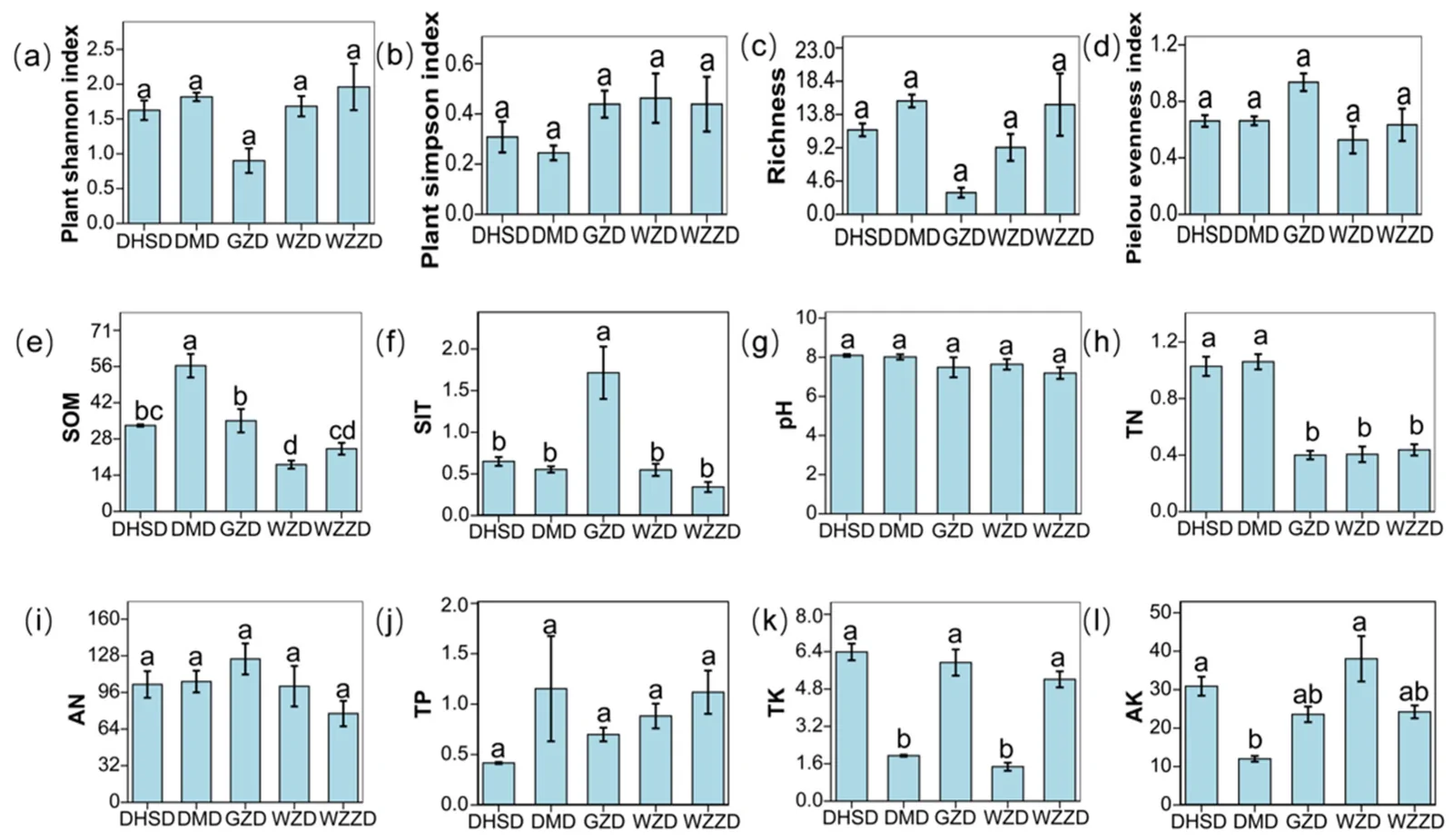
Soil physicochemical properties and plant diversity across the five oceanic islands in the northern South China Sea. Panels show (**a**–**d**) plant diversity indices and (**e**–**l**) soil properties. Abbreviations: SOM, soil organic matter; SIT, soil salt content; TN, total nitrogen; AN, available nitrogen; TP, total phosphorus; TK, total potassium; AK, available potassium. DHSD, Da-Han-San-Dun Island; DMD, Da-Miao-Dun Island; GZD, Gan-Zhe Island; WZD, Wei-Zhou Island; WZZD, Wu-Zhi-Zhou Island. Different lowercase letters indicate significant differences among islands (*p* < 0.05).

Soil physicochemical properties differed among the five oceanic islands (Figure 2). Soil organic matter was higher on Da-Miao-Dun Island than on the other four oceanic islands, and soil salinity was higher on Gan-Zhe Island than on the other oceanic islands. Soil total nitrogen was significantly higher on Da-Han-San-Dun and Da-Miao-Dun Islands than on Gan-Zhe, Wei-Zhou, and Wu-Zhi-Zhou Islands. Soil total potassium was significantly higher on Da-Han-San-Dun, Gan-Zhe, and Wu-Zhi-Zhou Islands than on Da-Miao-Dun and Wei-Zhou Islands. Available potassium was higher on Da-Han-San-Dun and Wei-Zhou Islands than on Da-Miao-Dun Island, whereas plant diversity indices did not differ significantly among oceanic islands.

Climatic–geographic variables also differed among oceanic islands (Table S1). Wei-Zhou Island had the largest area, the greatest distance from the mainland, and the highest evaporation and sunshine duration, whereas Wu-Zhi-Zhou Island had the highest wind speed. Gan-Zhe Island had the highest mean annual temperature, while Da-Miao-Dun Island had the highest mean annual precipitation (Table S1).

### 3.2. Bacterial and Fungal Diversity and Community Composition

**Figure 3:**
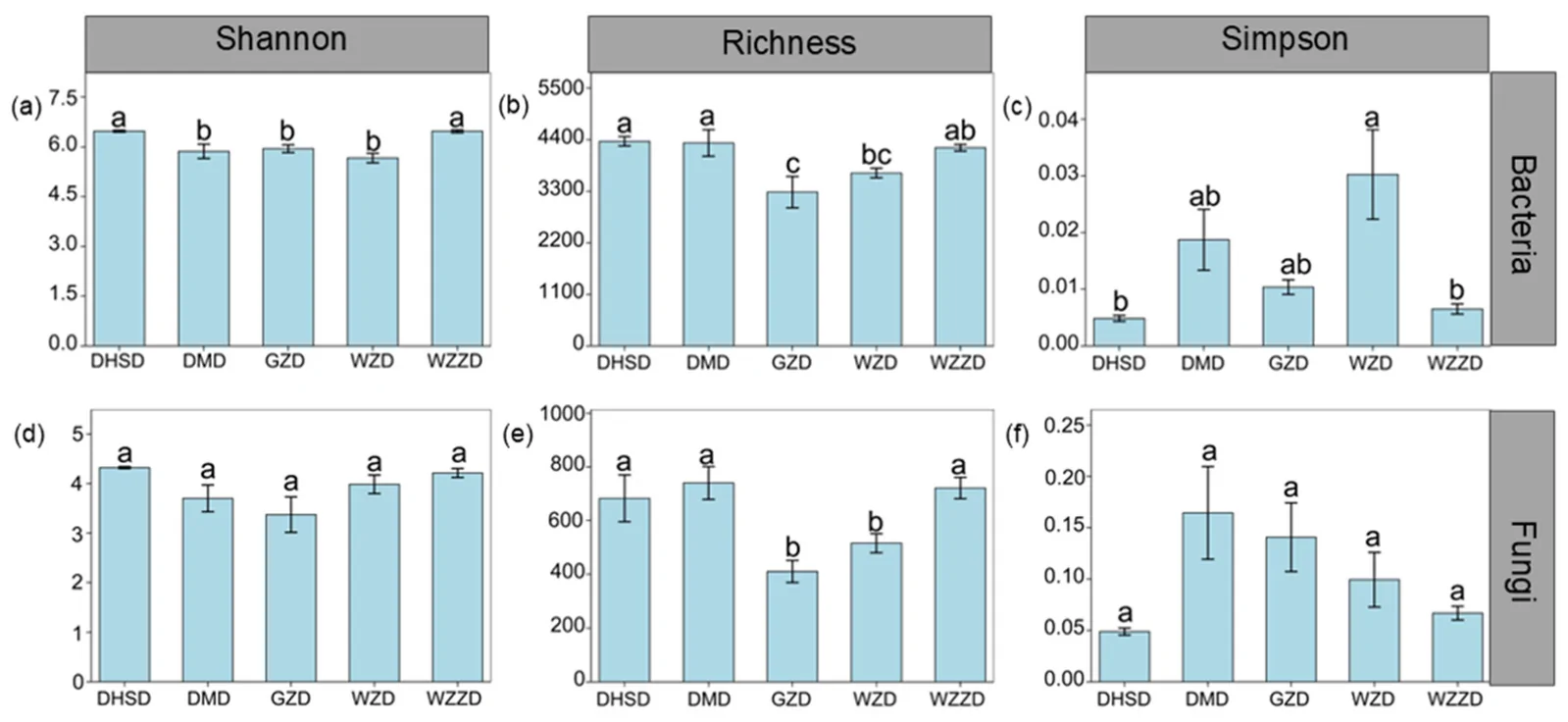
Bacterial and fungal diversity across five oceanic islands in the northern South China Sea. (**a**) Bacterial Shannon diversity; (**b**) bacterial ACE richness; (**c**) bacterial Simpson diversity; (**d**) fungal Shannon diversity; (**e**) fungal ACE richness; (**f**) fungal Simpson diversity. DHSD, Da-Han-San-Dun Island; DMD, Da-Miao-Dun Island; GZD, Gan-Zhe Island; WZD, Wei-Zhou Island; WZZD, Wu-Zhi-Zhou Island. Different lowercase letters indicate significant differences among islands (*p* < 0.05).

Bacterial Shannon diversity was higher on Da-Han-San-Dun and Wu-Zhi-Zhou Islands than on Da-Miao-Dun, Gan-Zhe, and Wei-Zhou Islands. Bacterial ACE richness was higher on Da-Han-San-Dun, Da-Miao-Dun, and Wu-Zhi-Zhou Islands than on Gan-Zhe Island, whereas fungal ACE richness was higher on Da-Han-San-Dun, Da-Miao-Dun, and Wu-Zhi-Zhou Islands than on Gan-Zhe and Wei-Zhou Islands. Bacterial Simpson diversity was higher on Wei-Zhou Island than on Da-Han-San-Dun and Wu-Zhi-Zhou Islands, whereas fungal Shannon and Simpson diversity did not differ significantly among islands (Figure 3). Significant island differences were detected for bacterial Shannon diversity (F_4,31_ = 9.614, *p* < 0.001, ω^2^ = 0.489), ACE richness (F_4,31_ = 6.924, *p* < 0.001, ω^2^ = 0.397), and Simpson diversity (F_4,31_ = 3.727, *p* = 0.014, ω^2^ = 0.233). For fungi, ACE richness differed significantly among islands (F_4,31_ = 8.748, *p* < 0.001, ω^2^ = 0.463), whereas Shannon diversity (F_4,31_ = 2.671, *p* = 0.0505, ω^2^ = 0.157) and Simpson diversity (F_4,31_ = 2.303, *p* = 0.081, ω^2^ = 0.126) did not.

**Figure 4:**
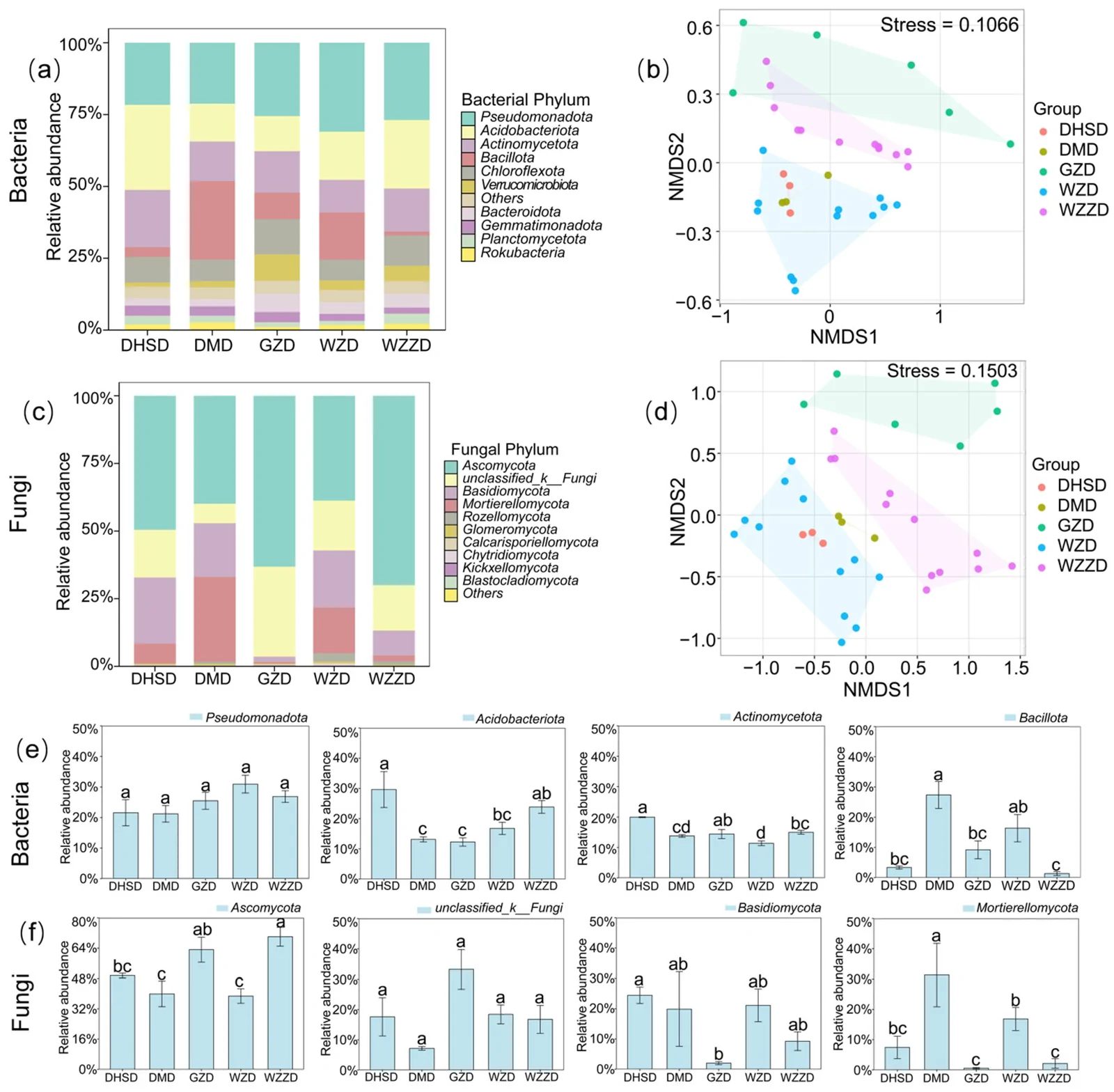
Bacterial and fungal community composition across five oceanic islands in the northern South China Sea. Panels show phylum-level relative abundance and NMDS ordinations for bacteria (**a**,**b**) and fungi (**c**,**d**), as well as the four most dominant phyla for bacteria (**e**) and fungi (**f**). Different lowercase letters indicate significant differences among islands (*p* < 0.05). DHSD, Da-Han-San-Dun Island; DMD, Da-Miao-Dun Island; GZD, Gan-Zhe Island; WZD, Wei-Zhou Island; WZZD, Wu-Zhi-Zhou Island.

NMDS ordinations showed island-associated variation in bacterial and fungal community composition, with stress values of 0.1066 and 0.1503, respectively (Figure 4b,d). PERMANOVA detected significant differences among islands for bacteria (pseudo-F_4,31_ = 2.649, R^2^ = 0.255, *p* < 0.001) and fungi (pseudo-F_4,31_ = 2.189, R^2^ = 0.220, *p* < 0.001). Multivariate dispersion also differed significantly among islands for bacteria (PERMDISP: F_4,31_ = 14.470, *p* < 0.001) and fungi (PERMDISP: F_4,31_ = 37.488, *p* < 0.001). The PERMANOVA results may therefore reflect differences in both group centroids and within-island dispersion.

At the phylum level, the relative abundance of *Acidobacteriota* was significantly higher on Da-Han-San-Dun Island than on Da-Miao-Dun, Gan-Zhe, and Wei-Zhou Islands, whereas *Actinomycetota* was significantly more abundant on Da-Han-San-Dun Island than on Da-Miao-Dun, Wei-Zhou, and Wu-Zhi-Zhou Islands. *Bacillota* was significantly more abundant on Da-Miao-Dun Island than on Da-Han-San-Dun, Gan-Zhe, and Wu-Zhi-Zhou Islands (Figure 4e). For fungi, *Ascomycota* was significantly more abundant on Wu-Zhi-Zhou Island than on Da-Miao-Dun and Wei-Zhou Islands, whereas *Mortierellomycota* was more abundant on Da-Miao-Dun Island than on the other islands (Figure 4f).

### 3.3. Bacterial and Fungal Co-Occurrence Networks

**Figure 5:**
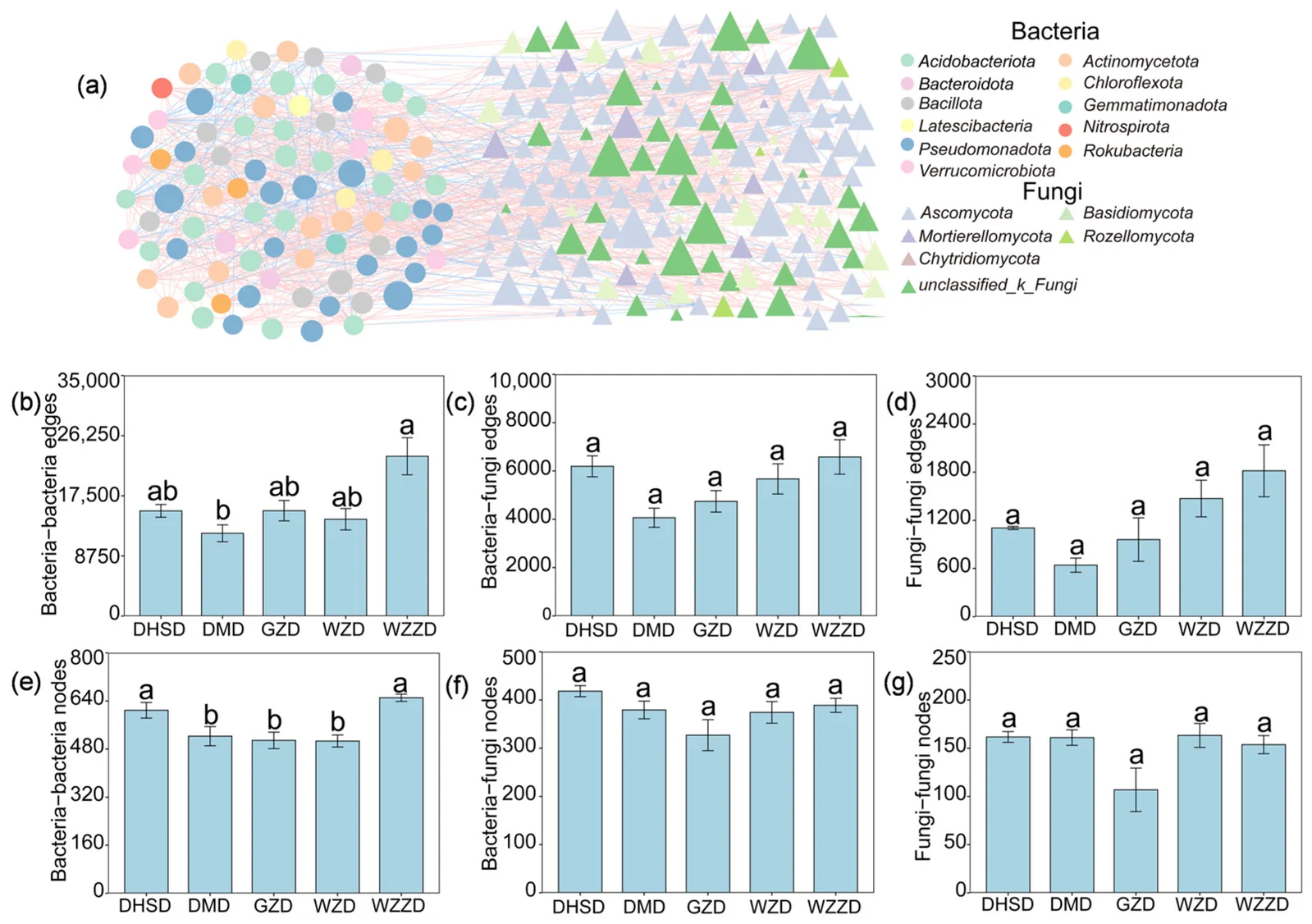
Co-occurrence patterns of bacterial and fungal communities across five nearshore islands. Panel (**a**) shows the integrated bacterial–fungal co-occurrence network, and panels (**b**–**g**) show the numbers of edges and nodes for bacteria–bacteria, bacteria–fungi, and fungi–fungi associations. Orange and blue edges represent positive and negative associations, respectively. Circles and triangles represent bacterial and fungal OTUs, respectively, and node colors indicate phylum-level taxonomic affiliation. DHSD, Da-Han-San-Dun Island; DMD, Da-Miao-Dun Island; GZD, Gan-Zhe Island; WZD, Wei-Zhou Island; WZZD, Wu-Zhi-Zhou Island. Different lowercase letters indicate significant differences among islands (*p* < 0.05).

Sample-level subnetworks extracted from the integrated co-occurrence network were used to compare bacterial and fungal co-occurrence patterns among islands (Figure 5). The number of bacteria–bacteria edges was significantly higher on Wu-Zhi-Zhou Island than on Da-Miao-Dun Island (Figure 5b). The number of bacteria–bacteria nodes was higher on Da-Han-San-Dun and Wu-Zhi-Zhou Islands than on Da-Miao-Dun, Gan-Zhe, and Wei-Zhou Islands (Figure 5e). In contrast, bacteria–fungi edges, fungi–fungi edges, bacteria–fungi nodes, and fungi–fungi nodes did not differ significantly among the five islands (Figure 5c,d,f,g).

Across all islands, *Actinomycetota* and *Pseudomonadota* accounted for relatively high proportions of bacteria-associated links, whereas *Ascomycota* accounted for a relatively high proportion of fungi-associated links (Figure 5).

### 3.4. Correlations Between Microbial Indices and Environmental Factors

**Figure 6:**
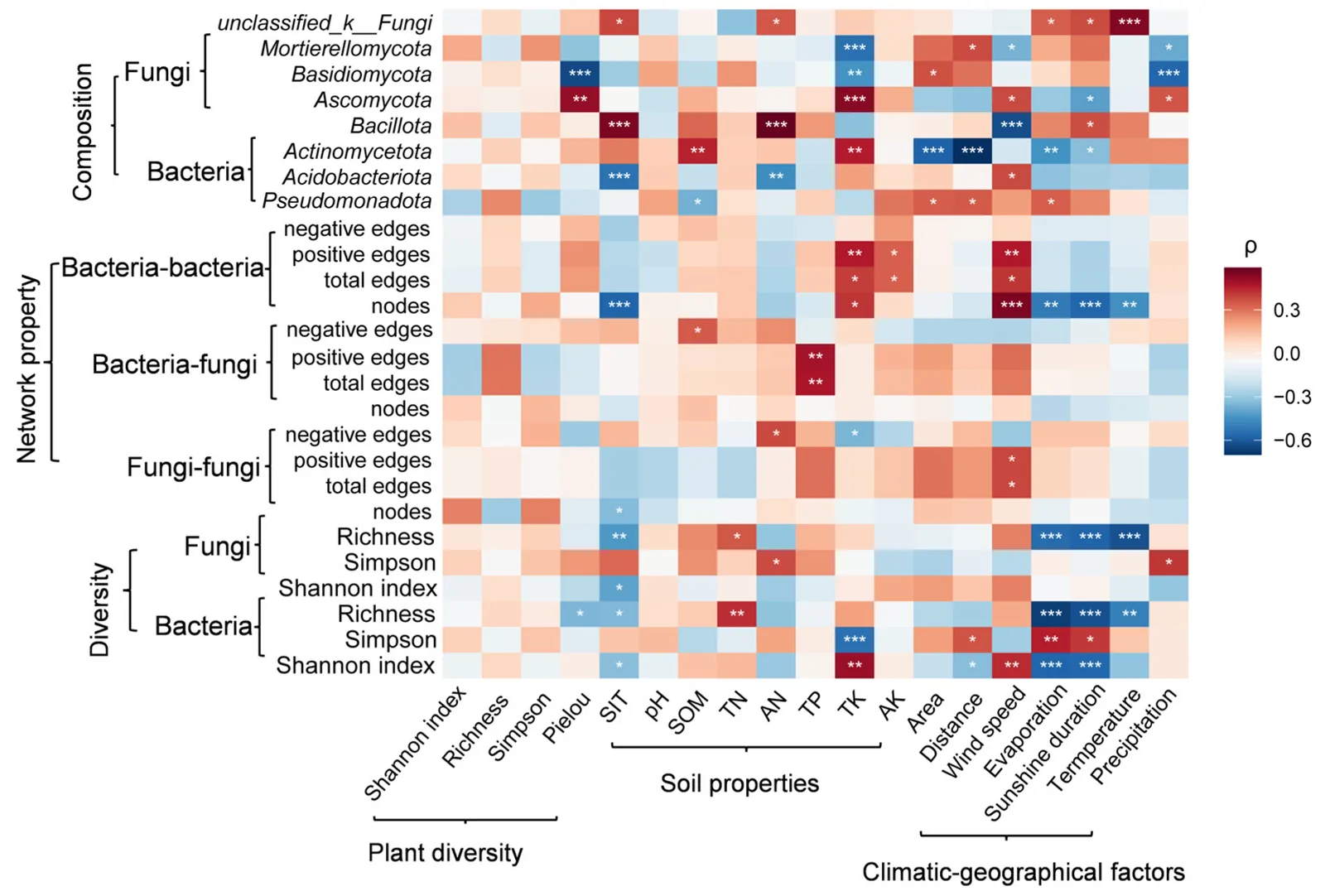
Spearman correlations among microbial indices, sample-level network properties, plant diversity, soil properties, and climatic–geographic factors. The color gradient represents the Spearman correlation coefficient (ρ), with red and blue indicating positive and negative correlations, respectively. Abbreviations: SIT, soil salt content; SOM, soil organic matter; TN, total nitrogen; AN, available nitrogen; TP, total phosphorus; TK, total potassium; and AK, available potassium. Climatic–geographic factors include island area, distance from the mainland, wind speed, evaporation, sunshine duration, mean annual temperature, and mean annual precipitation. Asterisks indicate unadjusted two-sided significance levels: * *p* < 0.05, ** *p* < 0.01, and *** *p* < 0.001. No multiple-testing correction was applied.

Spearman correlation analysis showed that the plant Shannon index was not significantly correlated with the microbial diversity, dominant-phylum relative-abundance, or sample-level network variables shown in Figure 6. Soil salinity was negatively correlated with the relative abundance of *Acidobacteriota*, bacterial ACE richness, fungal ACE richness, and the number of bacteria–bacteria nodes. In contrast, soil total potassium was positively correlated with bacterial Shannon diversity and with the numbers of total bacteria–bacteria edges, positive bacteria–bacteria edges, and bacteria–bacteria nodes. Among the climatic–geographic factors, evaporation, sunshine duration, and mean annual temperature were negatively correlated with bacterial and fungal ACE richness and the number of bacteria–bacteria nodes, whereas wind speed was positively correlated with the numbers of total bacteria–bacteria edges, positive bacteria–bacteria edges, and bacteria–bacteria nodes (Figure 6).

**Figure 7:**
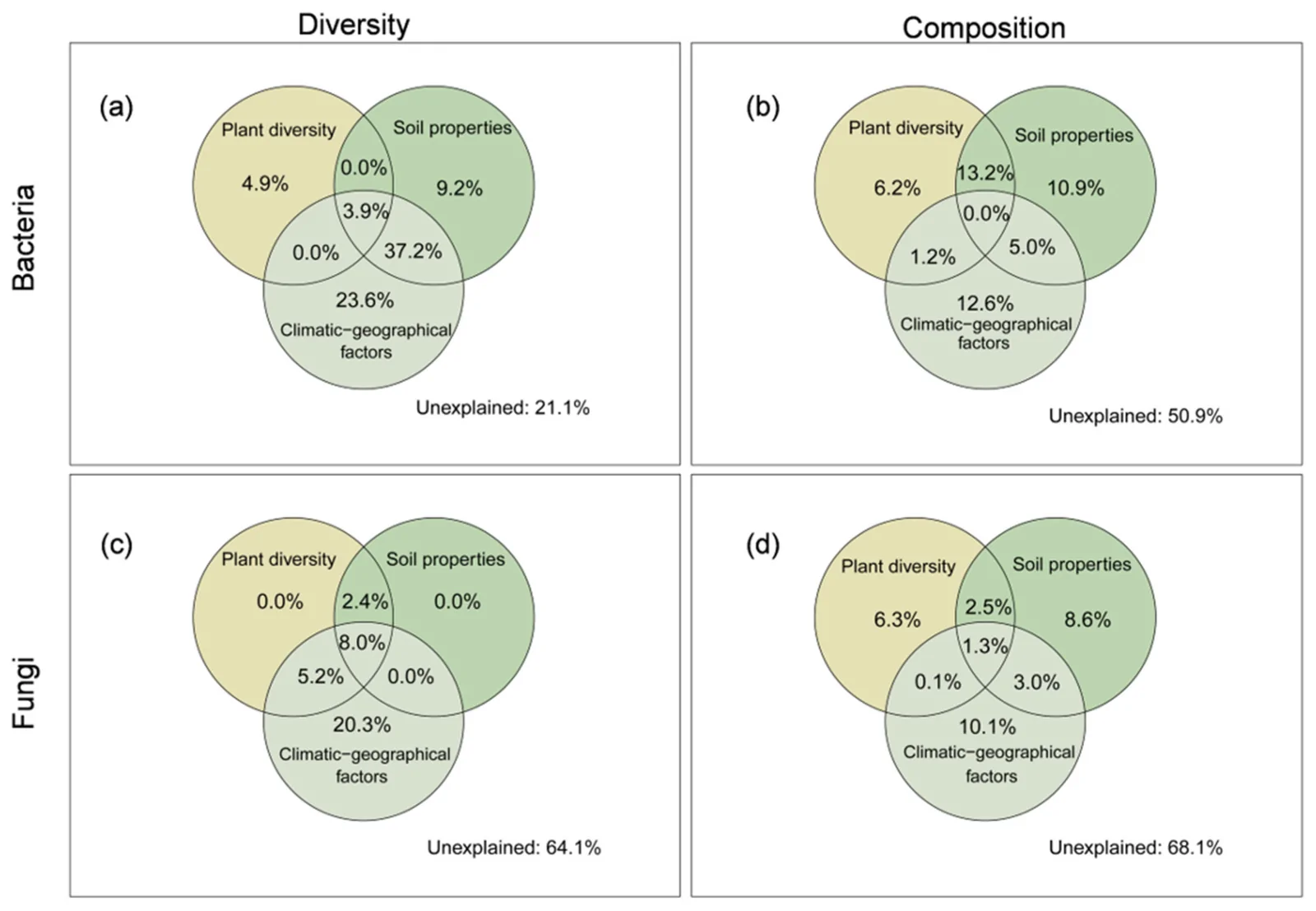
Variation partitioning of bacterial and fungal diversity and community composition associated with plant diversity, soil properties, and climatic–geographic factors. Panels show (**a**) bacterial alpha diversity, (**b**) bacterial community composition, (**c**) fungal alpha diversity, and (**d**) fungal community composition. Values indicate the percentage of variation explained by each factor group alone or in combination. Plant diversity was characterized by the Shannon index, Simpson index, species richness, and Pielou evenness index. Soil properties included soil pH, soil organic matter, total nitrogen, total phosphorus, total potassium, available nitrogen, available potassium, and soil salt content. Climatic–geographic factors included island area, distance from the mainland, wind speed, evaporation, sunshine duration, mean annual temperature, and mean annual precipitation.

Variation partitioning indicated that bacterial alpha-diversity variation was primarily associated with the shared fraction of soil physicochemical and climatic–geographic variables, which accounted for 37.2% of the variation, followed by the independent climatic–geographic fraction, which accounted for 23.6% (Figure 7a). For bacterial community composition, the shared plant–soil fraction, independent climatic–geographic fraction, and independent soil fraction accounted for 13.2%, 12.6%, and 10.9% of the variation, respectively (Figure 7b). For fungi, the independent climatic–geographic fraction accounted for the largest proportion of variation in both alpha diversity and community composition, explaining 20.3% and 10.1%, respectively (Figure 7c,d).

Together, the three predictor groups explained 78.9% of bacterial alpha-diversity variation, 49.1% of bacterial compositional variation, 35.9% of fungal alpha-diversity variation, and 31.9% of fungal compositional variation. The corresponding unexplained fractions were 21.1%, 50.9%, 64.1%, and 68.1%, respectively. These unexplained fractions may be associated with unmeasured soil moisture, microtopography, substrate quality, extracellular enzyme activity, microbial dispersal limitation, historical disturbance, and stochastic variation. VIFs ranged from 1.162 to 2.369 for the soil variables and from 6.181 to 97.973 for the plant-diversity indices, indicating low and high multicollinearity, respectively (Table S4). Because the climatic–geographic predictors were measured at the island level and only five islands were included, their individual effects were not interpreted separately.

## 4. Discussion

### 4.1. Associations of Soil Physicochemical and Climatic–Geographic Factors with Microbial Diversity Among Nearshore Islands

Microbial diversity varied significantly among oceanic islands, with these differences mainly linked to soil physicochemical properties and climatic–geographic stress rather than plant diversity indices. In this study, total nitrogen was positively associated with bacterial and fungal richness, while total potassium showed a positive relationship with bacterial Shannon diversity, indicating that higher nutrient availability was linked to greater microbial diversity across oceanic islands. This finding is consistent with previous studies showing that greater nutrient availability can enhance microbial diversity by improving resource supply and alleviating nutrient limitation [49,50]. In contrast, soil salinity was negatively associated with fungal richness, a pattern that was particularly evident on Gan-Zhe Island, where salinity was highest and fungal diversity was comparatively low. Similar studies have reported that increasing salinity can reduce microbial diversity by intensifying osmotic stress and excluding salt-sensitive taxa [51,52]. Together, these results suggest that nutrient availability was positively associated with bacterial richness and Shannon diversity, whereas salinity was negatively associated with fungal richness. Thus, the observed inter-oceanic island differences in bacterial and fungal diversity are consistent with our first hypothesis that microbial diversity would vary among the five oceanic islands.

Climatic–geographic factors further reinforced these inter-oceanic island differences. Evaporation and sunshine duration were negatively associated with bacterial richness, bacterial Shannon diversity, and fungal richness, whereas temperature was negatively associated with both bacterial and fungal richness. These patterns suggest that atmospheric drying processes reduce soil water availability, thereby limiting the persistence of microbial populations. Previous studies have similarly shown that drought and warming reduce microbial diversity, with bacterial communities often being more strongly suppressed, and our findings further support this pattern [53,54]. Therefore, microbial diversity across these islands was positively associated with nutrient availability and negatively associated with salinity and climatic drying. Correlation heatmaps further indicated that plant diversity contributed less to microbial diversity patterns than soil physicochemical properties and climatic–geographic factors.

### 4.2. Environmental Associations with Microbial Composition and Co-Occurrence Patterns

Differences in community composition among oceanic islands were accompanied by taxon-specific associations with salinity and moisture-related environmental conditions [52,55]. The relative abundance of *Acidobacteriota* was significantly negatively correlated with soil salinity, suggesting that salinity may act as an important environmental filter for bacterial communities. The relative abundance of *Acidobacteriota* was lower on Da-Miao-Dun, Gan-Zhe, and Wei-Zhou Islands than on Da-Han-San-Dun Island, whereas *Actinomycetota* showed a similar response pattern. By contrast, the relative abundance of *Bacillota* was relatively higher on Da-Miao-Dun Island, indicating a different response to oceanic-island environmental heterogeneity. Previous studies have shown that members of *Acidobacteriota* are often sensitive to saline or nutrient-stress conditions, whereas many members of *Bacillota* can persist under stressful environments because some lineages possess stress-resistant traits, including endospore formation [56,57]. For fungi, the relative abundance of *Ascomycota* was positively correlated with total potassium and negatively correlated with evaporation and sunshine duration, whereas the relative abundance of *Basidiomycota* was negatively correlated with precipitation. These contrasting responses indicate that fungal phyla may differ in their sensitivity to moisture and nutrient availability. Overall, variation in microbial community composition among oceanic islands was associated with taxon-specific patterns related to salinity, nutrient status, and climatic conditions. These taxon-specific shifts further support our first hypothesis that microbial community composition differs among oceanic islands, with these differences arising from contrasting responses of dominant bacterial and fungal phyla to environmental filtering [58]. These shifts in dominant bacterial and fungal groups may have implications for organic-matter decomposition and nutrient turnover on nearshore islands. Further functional measurements are needed to determine whether the observed taxonomic variation is accompanied by changes in microbial processes.

Soil physicochemical properties and climatic–geographic variables showed broader associations with microbial co-occurrence patterns than plant diversity [52,59], which showed no clear relationship with the edge or node variables in the heatmap. In our study, total potassium was positively correlated with bacteria–bacteria positive edges and bacteria–bacteria nodes, and total phosphorus was positively correlated with bacteria–bacteria edge numbers at the sample level. However, bacteria–fungi edge numbers did not differ significantly among oceanic islands, suggesting that island-level variation was less pronounced for cross-kingdom associations than for bacteria–bacteria associations. In contrast, soil salinity was significantly negatively correlated with the number of bacteria–bacteria nodes. Evaporation, sunshine duration, and temperature were also significantly negatively correlated with the number of bacteria–bacteria nodes. These patterns suggest that nutrient availability was positively associated with bacteria–bacteria edge and node numbers, whereas salinity and climatic drying were negatively associated with bacterial node numbers. Environmental conditions may therefore be related to differences in bacterial co-occurrence patterns among nearshore islands.

### 4.3. Relative Contributions of Plant Diversity, Soil Physicochemical Properties, and Climatic–Geographic Factors

Variation partitioning analysis revealed different patterns of explained variation for bacterial and fungal communities. Bacterial communities usually respond rapidly to changes in soil chemical properties, such as pH and nutrient availability, while also being sensitive to climatic controls on water and energy supply [60,61]. This finding was consistent with the relatively large fractions of bacterial diversity and compositional variation associated with soil physicochemical and climatic–geographic factors. In contrast, the lower explained variation for fungal diversity and community composition, together with the greater contribution of climatic–geographic factors, suggests that fungal communities were more strongly constrained by the broader environmental background. Previous studies have shown that fungal richness and community composition often retain strong climatic and spatial signals across broad scales, whereas their relationships with plant diversity are sometimes weaker than expected [62,63]. This pattern indicates that fungal distributions are influenced not only by soil resources, but also by biogeographic processes, dispersal limitation, and differences in substrate-use strategies among fungal taxa. Consistent with our second hypothesis, soil physicochemical and climatic–geographic factors accounted for larger fractions of microbial variation than plant diversity, although some of their explanatory fractions overlapped.

These findings highlight the relevance of soil salinity, nutrient status, and local climatic conditions for microbial biodiversity monitoring and soil management on nearshore islands. Nevertheless, this study was based on a single sampling campaign across five islands, with unequal numbers of plots among islands. Seasonal variation and the influence of human disturbance could therefore not be evaluated independently, and the significant differences in multivariate dispersion warrant caution when interpreting the PERMANOVA results. In addition, co-occurrence networks describe statistical associations rather than direct ecological interactions, and direct measurements of microbial functions were not included. Future studies combining multi-season sampling, broader island coverage, and measurements of microbial biomass, enzyme activity, and biogeochemical processes would help clarify the mechanisms underlying the observed patterns.

## 5. Conclusions

Soil bacterial and fungal communities exhibited distinct spatial patterns across five nearshore islands in the northern South China Sea. Bacterial alpha diversity varied more consistently among islands than fungal alpha diversity, whereas the composition of both bacterial and fungal communities differed among islands. Bacteria–bacteria edge numbers also differed among islands, while bacteria–fungi and fungi–fungi edge numbers showed no significant differences. Soil physicochemical and climatic–geographic factors accounted for larger fractions of microbial variation than plant diversity, highlighting the relevance of soil salinity, nutrient status, and climatic conditions to microbial variation on nearshore islands. These findings improve our understanding of bacterial and fungal biogeography in nearshore-island ecosystems and provide a basis for future microbial biodiversity monitoring and soil management.

## Data Availability

The original contributions presented in this study are included in the article/Supplementary Materials. Further inquiries can be directed to the corresponding author.

## Supplementary Materials

The following supporting information can be downloaded at: https://www.mdpi.com/article/10.3390/microorganisms14081838/s1. Table S1. Climatic-geographic information across five oceanic islands in the northern South China Sea; Table S2. Alpha-diversity results; Table S3. NMDS, PERMANOVA, and PERMDISP results; Table S4. Variance inflation factors for soil and plant-diversity variables used in the environmental analyses; Figure S1. Changes in the number of positive and negative edges in co-occurrence networks of bacterial and fungal communities across five oceanic islands.

## References

[1] Delavaux C.S., Crowther T.W., Bever J.D., Weigelt P., Gora E.M. (2024). Mutualisms weaken the latitudinal diversity gradient among oceanic islands. Nature.

[2] Hirschfeld M., Barnett A., Sheaves M., Dudgeon C. (2023). What Darwin could not see: Island formation and historical sea levels shape genetic divergence and island biogeography in a coastal marine species. Heredity.

[3] Barajas Barbosa M.P., Craven D., Weigelt P., Denelle P., Otto R., Diaz S., Price J., Fernandez-Palacios J.M., Kreft H. (2023). Assembly of functional diversity in an oceanic island flora. Nature.

[4] Whittaker R.J., Rigal F., Borges P.A., Cardoso P., Terzopoulou S., Casanoves F., Pla L., Guilhaumon F., Ladle R.J., Triantis K.A. (2014). Functional biogeography of oceanic islands and the scaling of functional diversity in the Azores. Proc. Natl. Acad. Sci. USA.

[5] Liu J., Chen M., Wang L., Liu T., Jin X., Yu F.H., Zhang Y. (2025). Habitat fragmentation differentially affects invasive and native plant diversity in a human-dominated wetland island system. Plant Divers..

[6] Li S.P., Wang P., Chen Y., Wilson M.C., Yang X., Ma C., Lu J., Chen X.Y., Wu J., Shu W.S. (2020). Island biogeography of soil bacteria and fungi: Similar patterns, but different mechanisms. ISME J..

[7] Wang P., Li S.P., Yang X., Zhou J., Shu W., Jiang L. (2020). Mechanisms of soil bacterial and fungal community assembly differ among and within islands. Environ. Microbiol..

[8] Xu M., Yang A., Yang X., Cao W., Zhang Z., Li Z., Zhang Y., Zhang H., You W., Yan E.R. (2023). Island area and remoteness shape plant and soil bacterial diversity through land use and biological invasion. Funct. Ecol..

[9] Zheng Y., Maitra P., Gan H.Y., Chen L., Li S., Tu T., Chen L., Mi X., Gao C., Zhang D. (2021). Soil fungal diversity and community assembly: Affected by island size or type?. FEMS Microbiol. Ecol..

[10] Cheng Y., Huang Y., Zhang R., Zhou S. (2025). Disentangling Drivers of Soil Bacterial and Fungal Diversity on Tropical Islands. Ecol. Evol..

[11] Kiesewetter K.N., Rawstern A.H., Cline E., Ortiz G.R., Santamaria F., Coronado-Molina C., Sklar F.H., Afkhami M.E. (2025). Microbes in reconstructive restoration: Divergence in constructed and natural tree island soil fungi affects tree growth. Ecol. Appl..

[12] Li S., Wang C., Yang S., Chen W., Li G., Luo W., Wei G., Chen C. (2023). Determining the contribution of microbiome complexity to the soil nutrient heterogeneity of fertile islands in a desert ecosystem. Sci. Total Environ..

[13] Guo J., Chu L., Ye X., King W.L., Shao J., Wang Z., Liu J., Chen C., Yu M. (2025). Low soil phosphorus and high symbiotic fungal richness inhibits plant aboveground biomass in fragmented forests in China. Commun. Biol..

[14] Zhao S., van der Heijden M.G.A., Banerjee S., Liu J.J., Gu H.D., Zhou N., Yin C.H., Peng B., Liu X., Wang B.Z. (2024). The role of halophyte-induced saline fertile islands in soil microbial biogeochemical cycling across arid ecosystems. Commun. Biol..

[15] Lu Y., Lyu M., Xiong X., Deng C., Jiang Y., Zeng M., Xie J. (2023). Understory ferns promote the restoration of soil microbial diversity and function in previously degraded lands. Sci. Total Environ..

[16] Holm J.B., Paragkamian S., Humphreys M., Chatterjee A., Yarwood S., Gaimaro J., Stavroulaki M., Tsaparis D., Plaitis M., Kasapidis P. (2025). First island-wide, single-day soil collection study on Crete reveals environmental drivers of microbial diversity. Environ. Microbiome.

[17] Nogales M., Traveset A., Lopez H., Heleno R., Rodriguez-Echeverria S., Garcia R., Hervias-Parejo S. (2025). Disentangling small-island multilayer networks: Underlying ecological and evolutionary patterns. Ecology.

[18] Gillespie L.M., Hättenschwiler S., Milcu A., Wambsganss J., Shihan A., Fromin N. (2021). Tree species mixing affects soil microbial functioning indirectly via root and litter traits and soil parameters in European forests. Funct. Ecol..

[19] Maitra P., Hrynkiewicz K., Szuba A., Jagodzinski A.M., Al-Rashid J., Mandal D., Mucha J. (2024). Metabolic niches in the rhizosphere microbiome: Dependence on soil horizons, root traits and climate variables in forest ecosystems. Front. Plant Sci..

[20] Lian W.-H., Zhao W.-S., Wang P.-D., Han J.-R., Lu C.-Y., Hu C.-J., Shi G.-Y., Chen F., Dong L., Zhou T. (2025). Habitat island biogeography of mountaintop plant and soil microbiomes: Similar patterns driven by different mechanisms. Glob. Ecol. Conserv..

[21] Yang H., Yu X., Song J., Wu J. (2024). Artemisia smithii patches form fertile islands and lead to heterogeneity of soil bacteria and fungi within and around the patches in alpine meadows of the Qinghai-Tibetan Plateau. Front. Plant Sci..

[22] Domeignoz-Horta L.A., Cappelli S.L., Shrestha R., Gerin S., Lohila A.K., Heinonsalo J., Nelson D.B., Kahmen A., Duan P., Sebag D. (2024). Plant diversity drives positive microbial associations in the rhizosphere enhancing carbon use efficiency in agricultural soils. Nat. Commun..

[23] Zhou T., Liang G., Reich P.B., Delgado-Baquerizo M., Wang C., Zhou Z. (2024). Promoting effect of plant diversity on soil microbial functionality is amplified over time. One Earth.

[24] Wan R., Hu J., Xiao H., Li Z., Xu J., He B., Song L., Wang L., Peng J., Yu X. (2026). Functional trait diversity in overstorey and understorey increases microbial carbon use efficiency in a forest plantation soil. Soil Biol. Biochem..

[25] Lei J., Su Y., Jian S., Guo X., Yuan M., Bates C.T., Shi Z.J., Li J., Su Y., Ning D. (2024). Warming effects on grassland soil microbial communities are amplified in cool months. ISME J..

[26] Li L., Xu Q., Jiang S., Jing X., Shen Q., He J.S., Yang Y., Ling N. (2024). Asymmetric winter warming reduces microbial carbon use efficiency and growth more than symmetric year-round warming in alpine soils. Proc. Natl. Acad. Sci. USA.

[27] Qin S., Zhang D., Wei B., Yang Y. (2024). Dual roles of microbes in mediating soil carbon dynamics in response to warming. Nat. Commun..

[28] Knight C.G., Nicolitch O., Griffiths R.I., Goodall T., Jones B., Weser C., Langridge H., Davison J., Dellavalle A., Eisenhauer N. (2024). Soil microbiomes show consistent and predictable responses to extreme events. Nature.

[29] Dacal M., García-Palacios P., Asensio S., Wang J., Singh B.K., Maestre F.T. (2022). Climate change legacies contrastingly affect the resistance and resilience of soil microbial communities and multifunctionality to extreme drought. Funct. Ecol..

[30] Oram N.J., Praeg N., Bardgett R.D., Brennan F., Caruso T., Illmer P., Ingrisch J., Bahn M. (2025). Drought Intensity Shapes Soil Legacy Effects on Grassland Plant and Soil Microbial Communities and Their Responses to Future Drought. Glob. Change Biol..

[31] Canarini A., Fuchslueger L., Schnecker J., Metze D., Nelson D.B., Kahmen A., Watzka M., Potsch E.M., Schaumberger A., Bahn M. (2024). Soil fungi remain active and invest in storage compounds during drought independent of future climate conditions. Nat. Commun..

[32] Lavallee J.M., Chomel M., Alvarez Segura N., de Castro F., Goodall T., Magilton M., Rhymes J.M., Delgado-Baquerizo M., Griffiths R.I., Baggs E.M. (2024). Land management shapes drought responses of dominant soil microbial taxa across grasslands. Nat. Commun..

[33] Wang C., Kuzyakov Y. (2024). Mechanisms and implications of bacterial-fungal competition for soil resources. ISME J..

[34] Wang P., Li S.P., Yang X., Si X., Li W.J., Shu W., Jiang L. (2023). Spatial scaling of soil microbial co-occurrence networks in a fragmented landscape. mLife.

[35] Zhou S., Qin H., Liao R., Cheng Y. (2024). Habitat quality drives the species–area relationship of plants and soil microbes in an ocean archipelago. Oikos.

[36] Wu Y., Wang B., Wu L., Liu S., Yue L., Wu J., Chen D. (2022). Fifty-year habitat subdivision enhances soil microbial biomass and diversity across subtropical land-bridge islands. Front. Microbiol..

[37] Cheng Y., Zhang R., Liao R., Qin H., Zhou S. (2026). Taxonomic evenness–area relationships for plant and soil microbes on tropical islands: Patterns and potential mechanisms. Oikos.

[38] Zheng Y., Liu X., Cai Y., Shao Q., Zhu W., Lin X. (2022). Combined intensive management of fertilization, tillage, and organic material mulching regulate soil bacterial communities and functional capacities by altering soil potassium and pH in a Moso bamboo forest. Front. Microbiol..

[39] Meng T., Shi J., Zhang X., Zhao X., Zhang D., Chen L., Lu Z., Cheng Y., Hao Y., Zhao X. (2024). Slow-release nitrogen fertilizer application regulated rhizosphere microbial diversity to increase maize yield. Front. Plant Sci..

[40] Wang X., Feng J., Ao G., Qin W., Han M., Shen Y., Liu M., Chen Y., Zhu B. (2023). Globally nitrogen addition alters soil microbial community structure, but has minor effects on soil microbial diversity and richness. Soil Biol. Biochem..

[41] Wang S., Wu Q.L., Li H., He R., Jiao C., Qin M., Deng Y., Zhang G., Zhao D., Zeng J. (2025). Bioclimatic zonation and spatial-scale dependence of lacustrine microbial assemblages. Sci. Bull..

[42] Du M., Xue P., Minasny B., McBratney A., Tang Y. (2026). Soil bacterial, fungal, and protistan assembly processes across a 1300 km climate and land-use transect. Catena.

[43] Oram N.J., Brennan F., Praeg N., Bardgett R.D., Illmer P., Ingrisch J., Bahn M. (2025). Plant community composition and traits modulate the impacts of drought intensity on soil microbial community composition and function. Soil Biol. Biochem..

[44] Zhang E., Wong S.Y., Czechowski P., Terauds A., Ray A.E., Benaud N., Chelliah D.S., Wilkins D., Montgomery K., Ferrari B.C. (2024). Effects of increasing soil moisture on Antarctic desert microbial ecosystems. Conserv. Biol..

[45] Wang B., Wang S., Wu L., Wu Y., Wang S., Bai Y., Chen D. (2024). Temporal asynchrony of plant and soil biota determines ecosystem multifunctional stability. Glob. Change Biol..

[46] Li Z., Duan X., Guo X., Gao W., Li Y., Zhou P., Zhu Q., O’Donnell A.G., Dai K., Wu J. (2024). Microbial metabolic capacity regulates the accrual of mineral-associated organic carbon in subtropical paddy soils. Soil Biol. Biochem..

[47] Gardes M., Bruns T.D. (1993). ITS primers with enhanced specificity for basidiomycetes-application to the identification of mycorrhizae and rusts. Mol. Ecol..

[48] Oren A., Garrity G.M. (2021). Valid publication of the names of forty-two phyla of prokaryotes. Int. J. Syst. Evol. Microbiol..

[49] Liu X., Han M., Zhang M., Wu C., Wang Z., Zhang Y., Chen Z. (2025). Fertilization managements mitigate microbial carbon and nitrogen limitations while preserving soil organic carbon in croplands compared to grasslands: A meta-analysis. J. Environ. Manag..

[50] Ortega R., Miralles I., Domene M.A., Meca D., Del Moral F. (2024). Ecological practices increase soil fertility and microbial diversity under intensive farming. Sci. Total Environ..

[51] Li C., Jin L., Zhang C., Li S., Zhou T., Hua Z., Wang L., Ji S., Wang Y., Gan Y. (2023). Destabilized microbial networks with distinct performances of abundant and rare biospheres in maintaining networks under increasing salinity stress. iMeta.

[52] Wang Z., Xia S., Bolan N., Li Q., Zhu Z., Yu B., Yang W., Fan Y., Bian R., Liu X. (2025). Elevated salinity decreases soil multifunctionality by driving bacterial community structure and network complexity. Sci. Total Environ..

[53] Sun Y., Chen H.Y.H., Chen X., Hisano M., Chen X., Reich P.B. (2025). Rising global temperatures reduce soil microbial diversity over the long term. Proc. Natl. Acad. Sci. USA.

[54] Preece C., Verbruggen E., Liu L., Weedon J.T., Peñuelas J. (2019). Effects of past and current drought on the composition and diversity of soil microbial communities. Soil Biol. Biochem..

[55] Xu J., Chen L., Zhou T., Zhang C., Zhang J., Zhao B. (2025). Salinity-driven differentiation of bacterial and fungal communities in coastal wetlands: Contrasting assembly processes and spatial dynamics. Environ. Res..

[56] Dong Y., Chen R., Graham E.B., Yu B., Bao Y., Li X., You X., Feng Y. (2024). Eco-evolutionary strategies for relieving carbon limitation under salt stress differ across microbial clades. Nat. Commun..

[57] Sliti A., Singh V., Pande A., Shin J.-H. (2025). Soil holobiont interplay and its role in protecting plants against salinity stress. Pedosphere.

[58] Bai X., Zhang E., Wu J., Ma D., Zhang C., Zhang B., Liu Y., Zhang Z., Tian F., Zhao H. (2024). Soil fungal community is more sensitive than bacterial community to modified materials application in saline-alkali land of Hetao Plain. Front. Microbiol..

[59] Kerfahi D., Guo Y., Dong K., Wang Q., Adams J.M. (2024). pH is the major predictor of soil microbial network complexity in Chinese forests along a latitudinal gradient. Catena.

[60] Winterfeldt S., Cruz-Paredes C., Rousk J., Leizeaga A. (2024). Microbial resistance and resilience to drought across a European climate gradient. Soil Biol. Biochem..

[61] Riddley M., Hepp S., Hardeep F., Nayak A., Liu M., Xing X., Zhang H., Liao J. (2025). Differential roles of deterministic and stochastic processes in structuring soil bacterial ecotypes across terrestrial ecosystems. Nat. Commun..

[62] Abrego N., Furneaux B., Hardwick B., Somervuo P., Palorinne I., Aguilar-Trigueros C.A., Andrew N.R., Babiy U.V., Bao T., Bazzano G. (2024). Airborne DNA reveals predictable spatial and seasonal dynamics of fungi. Nature.

[63] Lu Y., Han J., Zheng S., Chen Y. (2025). Cross-scale drivers of soil fungal diversity in fragmented forests of southwestern China. Commun. Biol..

