# Supplementary material for: Variation in Soil Bacterial and Fungal Diversity, Community Composition, and Co-Occurrence Patterns Across Nearshore Islands in the Northern South China Sea
Source: Microorganisms. 2026 Aug 19;14(8):1838. doi: 10.3390/microorganisms14081838 (PMC13515505; doi:10.3390/microorganisms14081838)
Supplement: Supplementary file 1 [file microorganisms-14-01838-s001.zip › microorganisms-4470802-supplementary.pdf]

Supplementary Material

Variation in Soil Bacterial and Fungal Diversity, Community Composition, and Co-occurrence Patterns across Nearshore Islands in the Northern South China Sea

Jian Gong[a,b,c], Yechen Qiao[a, b, c] , Rui Pan[b,c,d], Hu Du[b,c], Xionghui Liao[b,c], Wanxia Peng[a,b,c*]

[a] Guangxi Key Laboratory of Environmental Processes and Remediation in Ecologically Fragile Regions, Guangxi Normal University, Guilin 541006, Guangxi, China

[b] Institute of Subtropical Agriculture, Chinese Academy of Sciences, Changsha 410125, China

[c] Huanjiang Observation and Research Station for Karst Ecosystem, Guangxi Key Laboratory of Karst Ecological Processes and Services, Institute of Subtropical Agriculture, Chinese Academy of Sciences, Huanjiang 547100, China

[d] Guangxi Key Laboratory of Forest Ecology and Conservation, State Key Laboratory for Conservation and Utilization of Subtropical Agro-Bioresources, School of Forestry, Guangxi University, Nanning 530004, China

[*]Corresponding authors:

Wanxia Peng (E-mail: wxpeng@gxnu.edu.cn; Tel: 86-15802562131).

College of Environment and Resources, Guangxi Normal University, Guilin 541004, China

List of additional Tables

Table S1. Climatic-geographic information across five oceanic islands in the northern South China Sea.

Table S2 Alpha-diversity results

Table S3 NMDS, PERMANOVA, and PERMDISP results

Table S4 Variance inflation factors for soil and plant-diversity variables used in the environmental analyses.

Table S1 Climatic-geographic information across five oceanic islands in the northern South China Sea.

**Treatments**	**Area (km^2)**	**Distance (km)**	**Wind speed (m/s)**	**Evaporation (mm)**	**Sunshine duration (h)**	**Temperature (℃)**	**Precipitation (mm)**
DHSD	1.16	0.84	24.14	1216.25	1921.50	24.17	1485.00
DMD	0.62	1.19	20.56	1183.05	1613.10	22.73	2590.65
GZD	1.06	1.16	25.17	1326.80	2009.55	25.20	2525.50
WZD	24.74	37.16	37.89	1529.10	2145.40	23.51	1731.40
WZZD	4.80	2.76	45.86	1301.80	1663.05	22.92	2108.75

Table S2 Alpha-diversity results

Community	Index	df	F	P	(ω^2)
Bacteria	Shannon	4, 31	9.614	<0.001	0.489
Bacteria	ACE richness	4, 31	6.924	<0.001	0.397
Bacteria	Simpson	4, 31	3.727	0.014	0.233
Fungi	ACE richness	4, 31	8.748	<0.001	0.463
Fungi	Shannon	4, 31	2.671	0.0505	0.157
Fungi	Simpson	4, 31	2.303	0.081	0.126

Table S3 NMDS, PERMANOVA and PERMDISP

Community	NMDS	PERMANOVA			PERMDISP	
	stress	pseudo-F	R^2	P	F	P
Bacteria	0.1066	2.649	0.255	<0.001	14.470	<0.001
Fungi	0.1503	2.189	0.220	<0.001	37.488	<0.001

Table S4 Variance inflation factors for soil and plant-diversity variables used in the environmental analyses.

Variable	VIF
SIT	1.874
pH	2.369
SOM	2.098
TN	1.672
AN	2.327
TP	1.162
TK	1.622
AK	1.312

Variable	VIF
Shannon index	97.973
Richness	63.190
Simpson	19.648
Pielou	6.181

List of additional Figures

Figure S1 Changes in the number of positive and negative edges in co-occurrence networks of bacterial and fungal communities across five oceanic islands.

DHSD, Da-Han-San-Dun Island; DMD, Da-Miao-Dun Island; GZD, Gan-Zhe Island; WZD, Wei-Zhou Island; WZZD, Wu-Zhi-Zhou Island. Different lowercase letters indicate significant differences among islands ($p < 0.05$).

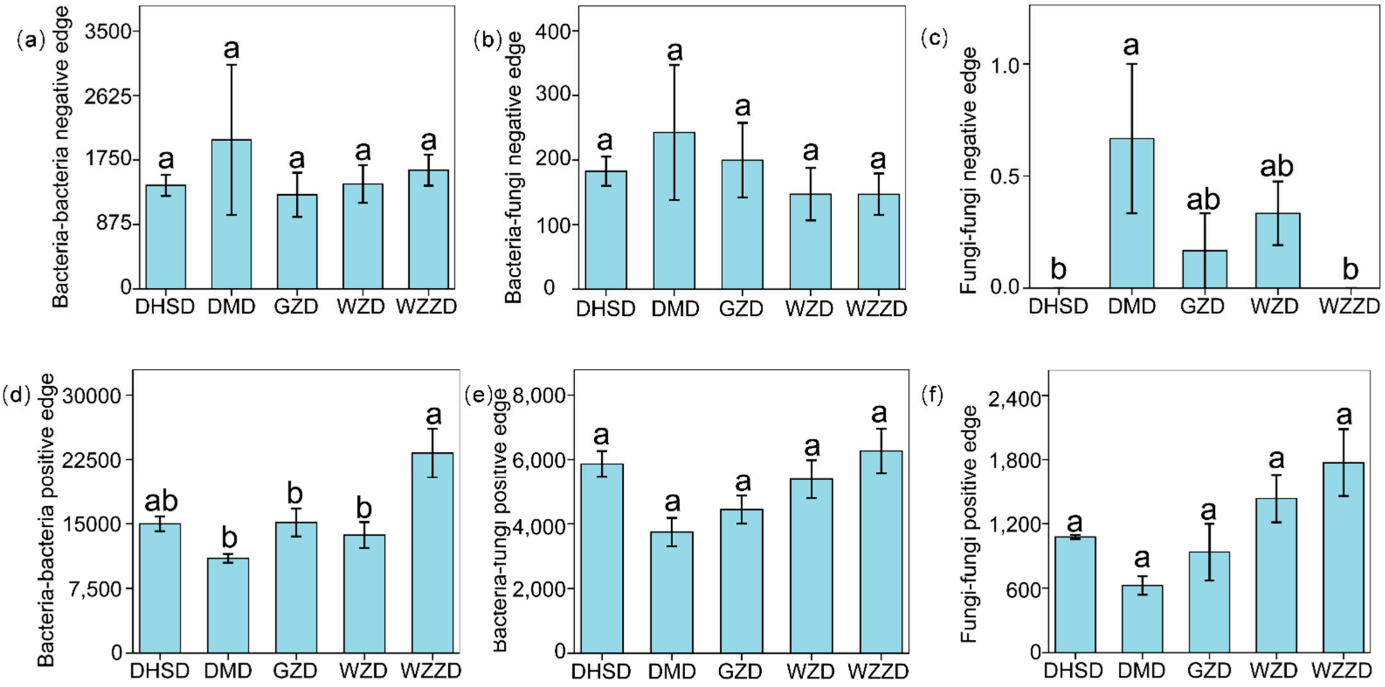

Figure S1 Changes in the number of positive and negative edges in co-occurrence networks of bacterial and fungal communities across five oceanic islands.

DHSD, Da-Han-San-Dun Island; DMD, Da-Miao-Dun Island; GZD, Gan-Zhe Island; WZD, Wei-Zhou Island; WZZD, Wu-Zhi-Zhou Island. Different lowercase letters indicate significant differences among islands ($p < 0.05$).
